# Toward a preventive approach to prolonged grief disorder in palliative care: Insecure attachment moderates the impact of perceived support on the severity of symptoms

**DOI:** 10.1371/journal.pone.0266289

**Published:** 2022-12-01

**Authors:** Vittorio Lenzo, Alberto Sardella, Alessandro Musetti, Cristina Faraone, Maria C. Quattropani

**Affiliations:** 1 Department Educational Sciences, University of Catania, Catania, Italy; 2 Department of Clinical and Experimental Medicine, University of Messina, Messina, Italy; 3 Department of Humanities, Social Sciences and Cultural Industries, University of Parma, Parma, Italy; 4 Sisifo—Consortium of Social Cooperatives, Catania, Italy; University of Technology Sydney, AUSTRALIA

## Abstract

**Objective:**

This study aimed to investigate the relationships between perceived support at the time of assistance, insecure attachment (i.e., avoidance and anxious attachment), and prolonged grief symptoms in family caregivers of palliative care patients deceased for at least one year. We also investigated the moderating role of insecure attachment in the relationship between perceived support and the intensity of prolonged grief symptoms.

**Method:**

An exploratory cross-sectional design was used. A sample of 157 participants completed the Prolonged Grief Scale (PG-13) and the Attachment Style Questionnaire (ASQ).

**Results:**

Correlational analyses indicated that prolonged grief symptoms were positively correlated with Avoidance attachment but not with Anxious attachment. Perceived support was negatively correlated with both the Avoidance and Anxious attachment factors. Lastly, the two insecure attachment dimensions were moderately and positively correlated with each other. Results of moderation analysis showed that high Avoidance attachment moderated the effect of perceived family and social support on the intensity of prolonged grief symptoms among family caregivers of palliative home-care-assisted patients. Results also showed that the Anxious attachment factor had a significant effect on prolonged grief symptoms, even though the interaction with perceived support was not significant.

**Conclusions:**

Overall, these results underline that a high level of avoidance attachment may moderate the relationship between perceived support and the intensity of grief symptoms, thereby increasing the risk of developing a mental disorder. Interventions to prevent prolonged grief disorder among family caregivers should take these findings into account.

## Introduction

Family caregivers of patients with advanced cancer face a stressful event that may have mental health consequences even after the loss [1]. Most of the bereaved regain quickly a normal functioning, while others may experience acute symptoms at the beginning [2]. Nonetheless, a pooled prevalence of 9.8 percent show symptoms of an abnormal grief reaction categorized as prolonged grief disorder (PGD) [3]. Proposed criteria for PGD included separation distress, together with cognitive, emotional, and behavioral symptoms [4]. Furthermore, the symptoms that characterize PGD are elevated at least 6 months after the loss and cause significant functional impairment. A central tenet of palliative care is to provide support during the patient’s illness [5], even though scant attention has been paid to what happens after the death of the loved one. If only a part of bereaved individuals shows an abnormal grief reaction, understanding the role of specific factors increasing the risk for prolonged grief disorder is challenging, even though fundamental for prevention. Worth noting, since the promising implications for implementation of the preventive approach, several studies have found significant relationships between complicated grief and a broad array of demographic and psychological variables, including social support and insecure attachment [6–8]. However, because of the limitations inherent to grief research, many unanswered questions remain about the interplay between potential predictor variables [9–11]. At the very least, it is reasonable to hypothesize that personality variables may play a moderating role in the relationship between perceived social support during the assistance and the outcome of bereavement. Turning to the psychological predictors, attachment theory has been considered a useful tool for understanding grief reactions [12, 13]. According to attachment theory, positive childhood experiences with caregivers are critical for developing secure internal working models of relationships and adequate emotion regulation abilities [14]. In the last decades, several studies have linked the quality of early attachment experiences with attachment relationships in adulthood [15, 16]. Perhaps not surprisingly then, people who did not attain a secure attachment, insofar their interactions with attachment figures during childhood have gone awry, are more likely to develop a mental disorder during adulthood [17]. The burgeoning literature on the person’s attachment style has focused attention on two relatively independent dimensions of insecure attachment named attachment-related anxiety and attachment-related avoidance, analogous to those observed in children [18, 19]. Whereas individuals with avoidant attachment tend to preserve emotional independence from adult attachment figures and distrust their goodwill, individuals with anxious attachment tend to worry that attachment figures will be inaccessible or elusive in times of distress [16]. The loss of a loved one represents a potentially overwhelming event that strongly strikes the attachment system and thereby can undermine the sense of security of the mourner [12]. Although feelings elicited by grief may vary hugely among bereaved individuals [20], its resolution hinges on the secondary attachment strategies of hyperactivation and deactivation [21]. These strategies are tantamount to the avoidance and anxious factors and virtually most of the studies have found a relationship with grief reactions [6]. To date, however, grief research leaves unsolved several crucial questions. A major research question regards the role of attachment in adjusting to the loss of a loved one. While attachment theory has posited that the lack of distress after the loss characterizing avoidant attachment style may lead to difficulties in the long run, it turned out that multiple pathways are possible, including seemingly maladaptive reactions [22]. Unfortunately, despite the relevance of understanding grief reactions and perhaps due to the difficulties in conducting research in this area, very few studies have investigated the interplay between insecure attachment factors and prolonged grief in the context of palliative home care [23].

**Fig 1:**
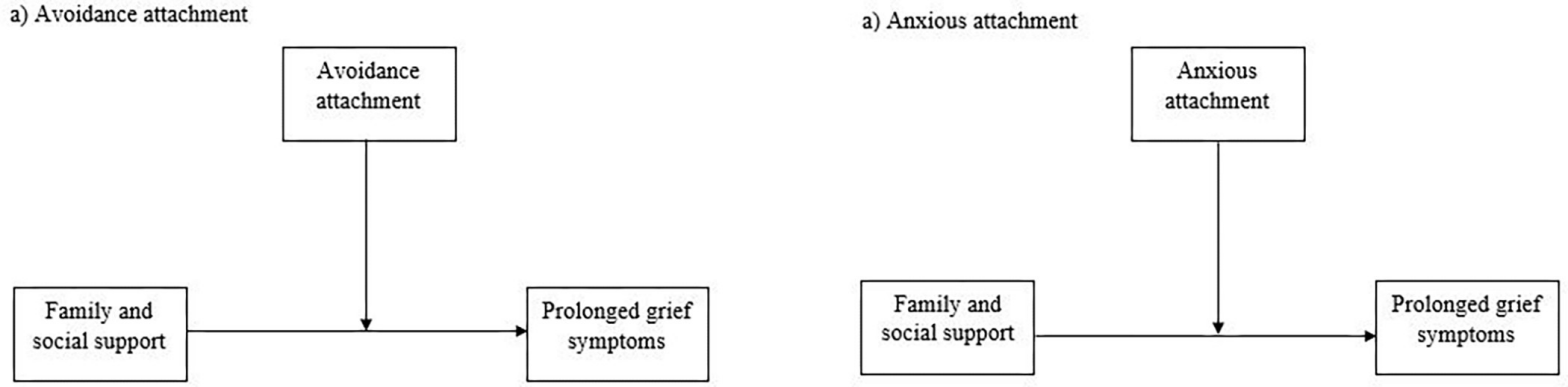
Conceptual model of the moderated role of Avoidance (a) and Anxious (b) attachment factors in the relationship between family/social support and prolonged grief symptoms.

Although it has been hypothesized that insecure attachment style may have a moderator effect, changing how family and social support is perceived [24], there is still a lack of studies in the context of grief reactions in palliative home care. Based on these premises, the present study has three main objectives. First, we sought to investigate the different intensity of prolonged grief symptoms on the basis of the kind of relationship with the deceased loved one. We hypothesized that widows experienced a higher intensity of symptoms than others. Second, we sought to examine the relationships between the perceived family and social support at the time of assistance, attachment insecurities (i.e., avoidance and anxious attachment factors), and the prolonged grief symptoms in a sample of family caregivers of palliative care patients deceased for at least one year. We hypothesized that avoidance and anxious attachment factors were positively correlated with each other and that, which in turn, were positively correlated with prolonged grief symptoms and negatively with perceived family and social support. Third, we sought to investigate the moderating role of avoidance and anxious attachment factors in the relationship between perceived family and social support at the time of assistance and grief intensity in family caregivers of palliative home care patients. The conceptual models representing the investigated variables in a moderate relationship are illustrated in Fig 1. We hypothesized that avoidance and insecure attachment factors are moderators of the relationship between perceived support and intensity of grief symptoms.

## Materials and methods

### Participants and procedure

This study is part of a larger research project named “Risk and protective factors for prolonged grief disorder in family caregivers of patients in palliative home care”. Participants were recruited between February 7, 2020 and November 9, 2021 among the caregivers of cancer patients who were assisted in three palliative home care services of three relatively small cities (Agrigento, Caltanissetta and Messina) in Sicily, Italy. Information about this study was provided by the clinical psychologists belonging to the three palliative home care services to potential participants after the death of their loved ones. Family caregivers who met the criteria and might have been interested in participating were contacted by the researchers. Participation was voluntarily, without any form of remuneration. The inclusion criteria were: being at least 18 years old, being able to give informed consent, and having lost a family member to cancer at least one year before enrolling in the study. Exclusion criteria were a pre-existing mental health disorder and/or taking psychotropic drugs at the time of participation in the study. All participants completed the survey anonymously and gave written informed consent before participating. Privacy of the participants was guaranteed in accordance with the European Union General Data Protection Regulation 2016/679. The research was conducted in accordance with the 1964 Declaration of Helsinki and its later amendments. This study was approved by the Research Ethics Committee for Psychological Research of the University of Messina (n. 38512). One hundred and fifty-nine subjects consented to participate and 157 completed the protocol.

### Measures

The participants completed a questionnaire including single-item questions on demographic (i.e., age, gender, and education), loss (i.e., year of bereavement, relationship with the patient, and work status), and family and social support variables. Regarding family and social support, the participants were asked to respond to the following question: “How much did you feel supported by your family and friends during the period of palliative care?” The response was rated on a 10-point Likert scale from 1 (“not at all”) to 10 (“very much”).

Moreover, the following two self-report measures were administered:

The Prolonged Grief Scale (PG-13) [25] is a self-report instrument to assess grief intensity and diagnose prolonged grief disorder related to the DSM-5 [4] and ICD-11 criteria [26]. The PG-13 consists of 11 items on a five-point Likert scale which assesses the severity/intensity of prolonged grief disorder. Indeed, each item refers to a symptom of prolonged grief disorder, such as cognitive, emotional, and behavioral symptoms. A total score consisting of the sum of all the items can be computed to obtain the intensity of the symptoms. Two more items are rated “yes/no” and permit to examine if the symptoms of separation distress are experienced more than once a day and if they are related to a significant diminution of social and occupational functioning. In the current study, the Italian version of PG-13 [27], which shows adequate psychometric properties, was used. The degree of reliability for this sample was excellent, with a Cronbach’s α of 0.88.

The Attachment Style Questionnaire (ASQ) [28] is a self-report instrument developed to evaluate attachment style factors. The 40 items of the questionnaire are scored on a six-point Likert scale which examines the following dimensions: Confidence (C); Discomfort with closeness (DwC); Relationship as secondary (RaS); Need for approval (NfA); Preoccupation with relationship (PwR). Following Hazan and Shaver’s [16] and Bartholomew’s [29] definition of attachment styles, the authors of ASQ endorsed insecure anxious/ambivalent attachment (through NfA and PwR) and insecure-avoidant attachment (through DwC and RaS). In the present study, the Italian version of ASQ [30], which shows acceptable psychometric properties, was adopted. For the purpose of this study, the higher-order two-factor structure of the ASQ, consisting of Avoidance and Anxious attachment factors, was used. The degree of reliability for this sample was excellent, with a Cronbach’s α ranging from 0.65 for Confidence to 0.76 for Relationship as secondary.

### Statistical analysis

The data were analyzed using SPSS v. 26 (IBM, Armonk, NY, USA) statistical software and the Process Macro for SPSS [31]. Descriptive results were described as frequencies (%), mean scores, and standard deviations. An Analysis of Variance (ANOVA) was performed to verify whether there were differences in the intensity of prolonged grief symptoms among caregivers based on the kind of relationship with the deceased loved one. The assumption of homogeneity of variance was verified using the Levene’s test. To achieve greater reliability of the results and to correct deviations from the normality of the sample distribution, the bootstrap procedure was carried out by 5000 simulation samples and with 5000 bootstrap replications for each sample (95% IC). Because of the heterogeneity of variance, Welch correction and post-hoc test using the Games-Howell technique were used.

Relationships between perceived support, PG-13, and ASQ-40 were performed with Pearson product-moment correlation coefficients. Subsequently, two distinct moderation analyses were performed to examine the role of Avoidance and Anxious attachment factors in moderating the effect of family and social support on the PG-13. Specifically, we considered the perceived support as an independent variable, the PG-13 as a dependent variable, and Avoidance and Anxious attachment factors as moderators. In each moderation analysis, the effect of interaction (family and social support x attachment insecurities factors) was decomposed through simple slope analysis at low (-1 *SD*), medium, and high (+1 *SD*) values of the moderator. We also used the Johnson-Neyman method to identify the exact value of the moderator at which the effect became significant. All the variables that define the product were centered, even though it was chosen to indicate raw values for the Johnson-Neyman solution and the figure for better clarity in understanding the results.

## Results

### Demographic and loss characteristics of the sample

The demographic and loss characteristics of the sample are shown in Table 1. The final sample consisted of 157 subjects who ranged in age from 18 to 81 years (*M* = 43.50 *±* 14.04). Most of the participants were female (77.1*%*), had a high school diploma (42*%*), and worked after loss (59.87*%*). Among participants, 52.3*%* were sons or daughters of the deceased and 57.3*%* were the main caregiver, while the time since the loss was on average 3.59 years (*SD* = 4.92; *Median* = 2).

**Table 1:** Demographic and loss characteristics of the sample (*N* = 157).

Characteristics	*n (%)*	*M*	*SD*
**Age (in years)**		43.50	14.04
**Gender**			
Male	36 (22.9)		
Female	121 (77.1)		
**Education**			
Primary or middle school diploma	34 (21.7)		
High school diploma	66 (42)		
Graduate	57 (36.3)		
**Work after loss**			
Yes	94 (59.87)		
No	63 (40.13)		
**Relation with the deceased loved one**			
Son or daughter	82 (52.3)		
Nephew	32 (20.4)		
Spouse	15 (9.5)		
Other (for example, brother-in-law)	28 (17.8)		
**Main caregiver**			
Yes	90 (57.3)		
No	67 (42.7)		
**Time since the loss (years)**		3.59	4.92

*Note*. The median for time since loss = 2 years.

### Relations with the deceased loved one and intensity of prolonged grief symptoms

Results of ANOVA showed that there were significant differences between the groups in the intensity of prolonged grief symptoms (Welch’s *F*(3, 46.953) = 13.417; *p* < 0.001). Table 2 illustrates the results of the Games-Howell post-hoc tests, interpreted through the bootstrap procedure. As for the PG-13 scores, results indicated that the mean score for spouses (*M* = 32.67; *SD* = 9.85) was significantly different from that for nephews (*M* = 21.69; *SD* = 5.51) as well as for others such as brother-in-law (*M* = 23.54; *SD* = 7.97). Results also indicated that the mean scores for both the nephews and brother-in-law were significantly different from those for the deceased loved one’s son or daughter (*M* = 29.37; *SD* = 8.61). The mean scores for spouses and sons or daughters were statistically similar.

**Table 2:** Games-Howell post-hoc test for intensity of prolonged grief symptoms based on the deceased loved one (*N* = 157).

Groups (based on the relations with the deceased loved one)		Difference mean (I-J)		Bootstrap	
			Standard Error	CI 95%	
				Lower	Upper
**Spouse (n = 15)**	Son or daughter	3.30	2.71	-2.27	8.46
	Nephew	10.98*	2.72	5.48	16.18
	Others	9.13*	2.93	3.19	14.58
**Son or daughter (n = 82)**	Spouse	-3.30	2.71	-8.46	2.27
	Nephew	7.68*	1.35	5.03	10.34
	Others	5.83*	1.75	2.40	9.28
**Nephew (n = 32)**	Spouse	-10.98*	2.72	-16.18	-5.48
	Son or daughter	-7.68*	1.35	-10.34	-5.03
	Others	-1.85	1.73	-5.24	1.49
**Others (n = 28)**	Spouse	-9.14*	2.93	-14.58	-3.19
	Son or daughter	-5.83*	1.74	-9.28	-2.40
	Nephew	1.85	1.73	-1.49	5.24

*Note*. Bootstrap based on 5000 simulated samples, *CI* = confidence interval

*p < .05.

### Correlational analysis between family and social support, intensity of prolonged grief symptoms, and attachment style factors

Table 3 displays descriptive statistics and correlation analyses. Results showed that family and social support was negatively and weakly correlated with both Avoidance (*r* = -.17; *p* < .05) and Anxious (*r* = -.25; *p* < .01) attachment factors. Results also showed that PG-13 was positively and weakly correlated with Avoidance attachment factor (*r* = .25; *p* < .01) but not with Anxious attachment factor and family/social support. Moreover, the time since the loss was weakly and negatively correlated with both the support (*r* = -.24; *p* < .01) and PG-13 (*r* = -.22; *p* < .01). Lastly, the two attachment insecurities factors were moderately and positively correlated (*r* = .43; *p* < .01).

**Table 3:** Descriptive and correlational analysis (*N* = 157).

Variable	*Min*	*Max*	*M*	*SD*	*Skew*	*Kurt*	1	2	3	4
1. Time since the loss (years)	1	28	3.59	4.92	3.11	0.39	-			
2. Family/social support	1	10	7.73	2.34	-1.17	1.04	-.24**	-		
3. Prolonged Grief Scale– 13	11	53	27.08	8.84	0.52	-0.16	-.22**	.08	-	
4. Avoidance attachment factor	32	93	56.31	11.22	0.32	0.19	.01	-.17*	.25**	-
5. Anxious attachment factor	19	81	47.03	12.68	0.25	-0.35	.06	-.25**	.18	.43**

*Note*. *Min* minimum value, *Max* maximum value, *M* mean, *SD* standard deviation, *Skew* skewness, *Kurt* kurtosis

**p < .01.

### Moderation of family and social support–prolonged grief symptoms association by attachment insecurity factors

Two separate moderation models were carried out to test whether Avoidance and Anxious attachment factors moderated the effect of perceived family and social support at the time of assistance on the intensity of prolonged grief symptoms.

**Fig 2:**
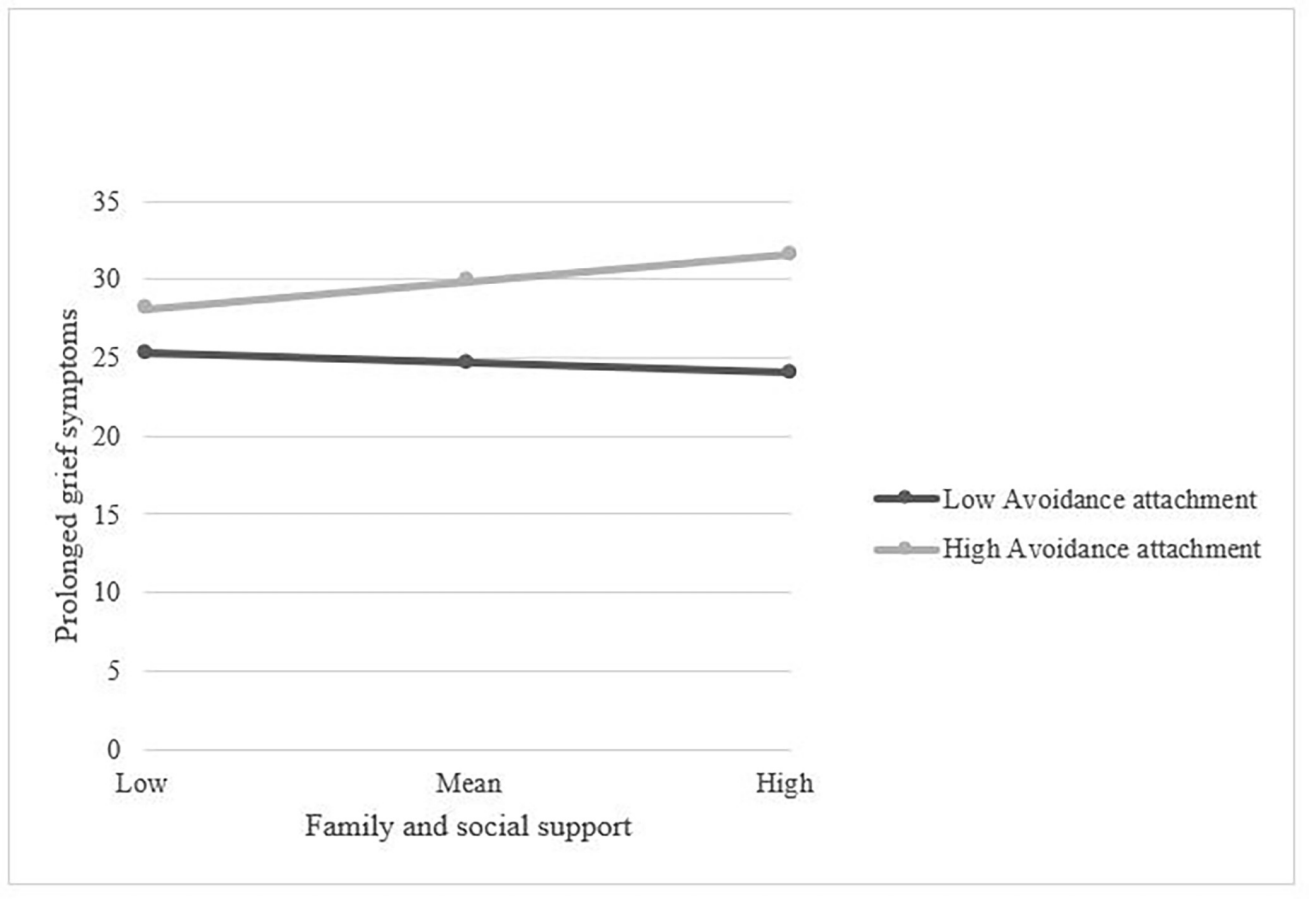
Interaction between perceived support and avoidance attachment for predicting prolonged grief symptoms among family caregivers.

Table 4 shows that Avoidance attachment factor (but not perceived support) had a significant effect on the prolonged grief symptoms (*B* = 0.23; *p <* .01). Moreover, family and social support predicted the severity of prolonged grief symptoms only at high levels of Avoidance attachment factor (*B* = 0.05; *p <* .05), but not at low or medium. Fig 2 represents the different slopes for the conditional effect of perceived family and social support on the prolonged grief symptoms highlighting the role of Avoidance attachment factor. The Johnson-Neyman method showed that for caregivers scoring on Avoidance attachment higher than 7.94, family and social support was related to the severity of prolonged grief symptoms.

**Table 4:** Avoidance attachment moderates the relationship between family/social support and intensity of prolonged grief symptoms.

	*B*	*SE*	*t*	95*%* CI
Intercept	27.28	0.68	39.00	[25.93, 28.63]
Family/social support	0.23	0.31	0.74	[-0.39, 0.85]
Avoidance attachment	0.23	0.06	3.72**	[0.11, 0.36]
Family/social support x Avoidance attachment	0.05	0.02	2.03*	[0.01, 0.09]
Regression Model *R*^*2*^ = .10**				
*Δ R*^*2*^ = .02*				
Conditional effects of Family/social support				
Low Avoidance attachment (-1*SD* = -11.22)	-0.28	0.47	-0.61	[-1.21, 0.64]
Medium Avoidance attachment (*M* = 0)	0.23	0.31	0.74	[-0.39, 0.85]
High Avoidance attachment (+1*SD* = 11.22)	0.75	0.33	2.28*	[0.10, 1.40]

*Note*. *CI =* confidence interval.

*p < .05; **p < .01.

Table 5 shows that Anxious attachment factor had a significant effect on the prolonged grief symptoms (*B* = 0.14; *p* < .01). As for the previous model, perceived support was not a significant predictor for prolonged grief symptoms. Furthermore, the interaction between the perceived support and Anxious attachment factor was not significant.

**Table 5:** Anxious attachment moderates the relationship between family/social support and intensity of prolonged grief symptoms.

	*B*	*SE*	*t*	95*%* CI
Intercept	27.33	0.71	38.61	[25.93, 28.73]
Family/social support	0.30	0.33	0.92	[-0.34, 0.95]
Anxious attachment	0.14	0.06	2.51**	[0.03, 0.25]
Family/social support x Anxious attachment	0.04	0.02	1.58	[-0.01, 0.08]
Regression Model *R*^*2*^ = .06*				

*Note*. Conditional effects of Family/social support are not present because the interaction (Family/social support x Avoidance attachment) was not significant, *CI* = confidence interval.

*p < .05

**p < .01.

## Discussion

This study aimed to explore the interplay between perceived support, insecure attachment factors, and the intensity of prolonged grief symptoms among caregivers who lost a family member to cancer. This general intent was addressed through two specific aims. First of all, we investigated the effect of the kind of relation with the deceased loved one on the intensity of prolonged grief symptoms. Our findings sustained the hypothesis that the spouses experienced higher intensity of symptoms than others such as nephews. Likewise, the mean scores for spouses were quite similar to those of the deceased loved one’s sons or daughters. Admittedly, since that Holmes and Rahe [32] detected the death of a spouse or a close family member as the two of the most stressful life events, our findings do not stand out. However, our main interest was to outline the psychological factors underlying grief reaction in the context of palliative care. For this reason, our second aim was to examine the correlational coefficients between family and social support during the period of assistance, avoidance and anxious attachment factors, and prolonged grief symptoms. As expected, we found a moderate and positive correlation coefficient between the two insecure attachment factors. This finding is coherent with previous research highlighting that avoidance attachment factor and anxious attachment factor are not incompatible [30]. Another finding was that support from loved ones and friends was inversely associated with both factors of insecure attachment. When considering this relationship, findings from past studies showed analogous results, even though none of the studies have considered bereaved subjects in the context of palliative care [33]. Worth noting, we remind that attachment style during adulthood may influence the extent to which individual considers support from family and social in general. Thus, family caregivers of patients assisted in palliative home care with insecure attachment style may perceive lower support or may repute it as not important. Nonetheless, our interest was also to investigate the relationship between insecure attachment and symptoms of prolonged grief. In addition to improving our understanding of the prevalence and characteristics related to the onset of prolonged grief disorder, findings from this field of research may be useful for its prevention. As revealed by our results, avoidant (but not anxious) attachment factor was related to the reported symptom of grief. Since that Deutsch [34] has introduced the term “Absence of Grief” for understanding a lack of conscious affect following a loss, the avoidance attachment style has been proposed to explain fewer symptoms of prolonged grief in bereaved people, even though empirical research has produced contradictory results [21]. Whatever the cause of such inconsistent results, a promising line of research has investigated not only the relationship between attachment and grief but also the role of some moderating variables [35]. In this vein, the third aim of this study was to verify if avoidance and anxious attachment factors impinge on the relationship between perceived support and symptoms of prolonged grief in family caregivers. The burgeoning literature has well demonstrated that cancer patients who perceived low social support showed higher levels of depression and a worse quality of life [36, 37]. However, as regards bereaved individuals, it is worth highlighting that the role of social support in protecting from adverse mental health outcomes remains ambiguous [8]. It has been argued that social support cannot sufficiently mitigate the loss of a loved one [38]. As depicted by our findings, there was not a direct effect of perceived support on the intensity of prolonged grief symptoms among the bereaved people composing our sample. For the sake of clarity, it is worth emphasizing that the low reported support may be reveal conflicting relations rather than the lack of supportive relationships [39], which in turn may be related to the functioning of the attachment system. Of particular interest in our study is that both avoidance and anxious attachment factors showed a direct effect on the reported symptoms of grief. Nonetheless, avoidance attachment seems to moderate the effect of perceived support on the outcome of bereavement. A central tenet of the Bowlby’s theory (1980) links the avoidant attachment to the absence of grief and, consequently, we are aware that our finding may engender perplexity. However, recent empirical evidence has depicted more nuances in the relationship between emotionally avoidant individuals and their maladaptive reaction to loss. In this research field, indeed, Bartholomew (1990) differentiate attachment avoidance into two attachment styles, that are named “fearful-avoidant” and “dismissing-avoidant”. In this vein, caregivers involved in this study might be characterized by higher levels of fearful-avoidant attachment. In contrast to dismissing-avoidant individuals tending to have less difficulty adjusting to loss, the fearful-avoidant ones may have more problems because they are hugely anxious about attachment concerns. In turn, fearful-avoidant individuals tend to organize their behavior in a defensive way aiming to allay their insecurities. Admittedly, they avoid emotionally investing in others due to the fear of being rejected. Hence, social and familiar support received during the assistance of their loved one is not effective in mitigating the prolonged grief symptoms after the loss. Actually, it can increase its intensity.

Likewise, how the support is perceived by the individual may hinge on its attachment style on the grounds that, in turn, it influences the extent to which he or she considers essential support from family members and friends. Turning to the two dimensions that characterize insecure attachment (i.e., avoidance and anxious), Mikulincer [21] argued that their balancing weight is paramount to coping with grief. Hence, taken together with results of correlational analysis, our findings may indicate an imbalance in this dynamic, even though more research focusing on bereaved individuals who lost a family member in the context of palliative care is needed. Indeed, knowledge on the factors increasing the risk of prolonged grief disorder in the context of palliative care would have practical value for its prevention.

Although our findings increase the understanding of the risk of prolonged grief disorder in the context of palliative care, there are also some limitations that future research should take into account. First, this study adopted a cross-sectional design that did not allow us to conclude without uncertainty for a causal relationship between the observed variables. Moreover, it is not possible to entirely exclude that the ASQ scores may fluctuate with significant life events such as the death of the loved one, even though attachment style dimensions tend to be stable over time [40]. Future studies adopting a longitudinal design would better account for the long-lasting role of insecure attachment style on the association between perceived support and prolonged grief symptoms. Longitudinal studies would also verify any possible fluctuation in relation to significant life events including the death of a loved one. Second, the oversampling of certain characteristics (i.e., the female gender) among the participants as well as some variables not normally distributed (i.e., the time since death) may have influenced the results and whereby may be not generalizable to other bereaved individuals. Nonetheless, some characteristics of our sample such as a majority of female gender can be considered as representative of family members assisting their loved ones with a terminal illness. Third, the exclusion of participants with pre-existing mental health disorder and/or taking psychotropic drugs may have limit the generalizability of our findings. Although the mean score of the PG-13 was similar to what other studies found [41, 42], we may have included only stable bereaved family caregivers who did not require medication at time of study participation.

## Conclusions

In sum, our results suggested that avoidance attachment behaves as a moderator of the relationship between perceived social support and symptoms of prolonged grief disorder among bereaved caregivers. Despite both avoidant and anxious attachment showed an effect on the intensity of prolonged grief symptoms, the interaction with perceived support was significant only for the avoidance attachment dimension. These results highlight the specificities among people with different insecurity attachment facing the loss of a loved one. Indeed, support from family members and friends may be helpful, even though high levels of avoidant attachment may interfere with the grief resolution and worsen the outcomes. Specialists and researchers within the field of palliative care may find useful our findings when assessing and preventing prolonged grief disorders among bereaved individuals.

## Supporting information

S1 Dataset (XLSX)
